# The relationship between lymphovascular invasion and angiogenesis, hormone receptors, cell proliferation and survival in patients with primary operable invasive ductal breast cancer

**DOI:** 10.1186/1472-6890-13-31

**Published:** 2013-11-25

**Authors:** Zahra MA Mohammed, Donald C McMillan, Joanne Edwards, Elizabeth Mallon, Julie C Doughty, Clare Orange, James J Going

**Affiliations:** 1 Academic Unit of Surgery, College of Medical, Veterinary and Life of Sciences, Royal Infirmary, University of Glasgow, Glasgow G31 2ER, UK; 2 University Departments of Pathology, Faculty of Veterinary Medicine, Omar Almukhtar University, Al bayda, PO Box 919, Libya; 3 Unit of Experimental Therapeutics, Institute of Cancer, College of Medical, Veterinary and Life Sciences, Royal and Western Infirmary, University of Glasgow, Glasgow G11 6NT, UK; 4 University Department of Pathology, College of Medical, Veterinary and Life of Sciences, Royal and Western Infirmaries, University of Glasgow, Glasgow G11 6NT, UK; 5 Department of Surgery, Western Infirmary, Glasgow G11 6NT, UK

**Keywords:** Primary invasive breast cancer, Prognostic factors, Lymphovascular invasion, Angiogenesis, Survival

## Abstract

**Background:**

Several well-established tumour prognostic factors are used to guide the clinical management of patients with breast cancer. Lymphovascular invasion and angiogenesis have also been reported to have some promise as prognostic factors. The aim of the present study was to examine the prognostic value of tumour lymphovascular invasion and microvessel density compared with that of established prognostic factors in invasive ductal breast cancer.

**Methods:**

In addition to hormone receptor status and Ki-67 proliferative activity, lymphovascular invasion and microvessel density and their relationship with survival were examined in patients with invasive ductal breast cancer. Full sections and tissue microarrays (n = 384 patients) were utilised to assess these factors and were scored by appropriate methods.

**Results:**

On univariate analysis tumour size (*P* < 0.05), lymph node involvement (*P* < 0.01), lymphovascular invasion (*P* < 0.05), microvessel density (*P* < 0.05) and local- regional treatment (*P* < 0.01) were associated with poorer survival in ER negative tumours. On multivariate analysis in ER negative tumours lymph node involvement (*P* < 0.01) and local- regional treatment (*P* < 0.05) were independently associated with poorer cancer-specific survival. On univariate analysis tumour grade (P < 0.05), lymph node involvement (*P* < 0.001), HER-2 (*P* < 0.05), Ki-67 (*P* < 0.01) and lymphovascular invasion (*P* < 0.001) were associated with poorer survival in ER positive tumours. On multivariate analysis lymph node involvement (*P* < 0.001), Ki-67 (*P* < 0.001) and lymphovascular invasion (*P* < 0.05) were independently associated with poorer cancer-specific survival in ER positive tumours.

**Conclusion:**

Lymphovascular invasion but not microvessel density was independently associated with poorer survival in patients with ER positive but not ER negative invasive ductal breast cancer.

## Background

Breast cancer is the commonest cancer and the leading cause of cancer death in women accounting for 22% of all female cancers [1]. Although approximately 80% of the 42,000 women in the UK are diagnosed with breast cancer each year and survive at least five years [2], it is still the leading cause of cancer death in women.

Prognostic factors aid clinical decision making, treatment selection for individual patients and allow comparisons between groups of patients at risk of recurrence or death. Clinically useful prognostic/predictive factors should have biological relevance, be reproducible in different laboratories, be validated prospectively in large series of patients, be confirmed independently by other workers and have threshold levels that are already optimized [3].

With reference to breast cancer, well-established clinicopathological indicators of clinical outcome and response to therapy are age, histologic type, grade, tumour size, lymph node status and hormone receptor expression [4,5]. Expression of oestrogen receptor (ER) and progesterone receptor (PR) is associated with better survival and response to oestrogen competive agonists such as tamoxifen, independently of other variables [4,6-10]. More recently, human epidermal growth factor receptor 2 (HER-2) status has become an established clinicopathological indicator of breast cancer clinical outcome and response to therapy [4,5]. Proliferation is recognised to be a key feature of tumour progression and is now widely estimated by the nuclear antigen Ki-67, which is tightly linked to the cell cycle. Several recent studies have reported an association between higher Ki-67 proliferative activity and poorer recurrence-free [11-13] and cancer-specific survival [14-16].

Lymphovascular invasion is a crucial step in the complex process of tumour metastasis and an important criterion for further therapy. The presence of carcinoma cells in either lymphatic vessels (lymphatic invasion), blood vessels (vascular invasion) or both (lymphovascular invasion) is a significant prognostic factor in invasive breast cancer, with respect to local and distance recurrence [17-22] and poorer survival [19-25]. At the St. Gallen meeting in 2005, lymphovascular invasion was recognised as a prognostic factor for node-negative patients [26]. Node-negative patients with lymphovascular invasion had higher breast cancer mortality rate (53%) compared with patients with no lymphovascular invasion (29%) [27]. Lymphovascular invasion is also associated with other strongest prognostic factors including tumour size, grade and loco- regional lymph node involvement [21,27,28].

The role of angiogenesis (development of new capillaries from pre-existing vessels) in the growth of solid tumours is recognized [29]. Furthermore, it is a vital not only for the development and progression of primary carcinomas but also for invasion and metastasis of solid tumours [30]. Accumulating evidence indicate that progressive tumour growth is dependent on angiogenesis [31] and several studies have reported an association between microvessel density and poorer recurrence- free [23,32-37], cancer- specific survival [16] and overall survival [16,23,32,34,35,38]. Microvessel density is also associated with a poorer clinical response to chemotherapy [39] and to endocrine therapy [39,40]. On the other hand, some authors do not find any association between microvessel density and recurrence- free survival and overall survival [41,42] and one found a direct association between microvessel density and clinical response to chemotherapy [43]. To date there have been few studies that have directly compared these different approaches and their relationship with outcome. Moreover, it is not clear whether angiogenesis, as measured by microvessel density, may add additional prognostic information to established prognostic and predictive factors. Therefore, the aim of the present study was to examine the prognostic value of lymphovascular invasion and microvessel density and compare it with other well-established clinicopathological prognostic factors.

## Methods

Patients presenting with invasive breast cancer at Glasgow Royal and Western Infirmaries and Stobhill Hospital, all in the West of Scotland, between 1995 and 1998 and who had lymphovascular invasion assessed were studied (n = 384). Available clinicopathological data included age, histological tumour type, grade, tumour size, lymph node status, type of surgery and use of adjuvant treatment (chemotherapy, hormonal therapy and/or radiotherapy).

The study was approved by the Research Ethics Committee of the North Glasgow University Hospitals NHS Trust.

### Tissue micro array (TMA) construction

Tissue microarrays (TMA) were used in this study. In brief, each specimen had tumour rich area identified by a pathologist and 3 cores 0.6-mm^2^ tumour cores were used to construct the TMA [44].

### Assessment for tumour ER, PR and HER-2 statuses and Ki-67 proliferative index

Tissue microarrays (TMA) were utilized to assess Oestrogen (ER), progesterone (PR) status and HER-2 status as previously described [45,46] and to assess Ki-67 proliferative activity as previously described [47]. Also TMA were used to assess microvessel density by immunohistochemistry for CD34 +.

### Immunohistochemistry for microvessel density

In addition to TMA, Immunoreactivity to anti-CD34+ antibody was determined in 33 cases by using full-face tissue sections. Freshly cut 2.5 µm thick breast cancer full-face tissue sections and TMAs were stained for CD34+. Sections and cores were dewaxed and rehydrated for antigen heat-retrieval in Tris EDTA buffer (pH 8), under pressure for 7 minutes. Endogenous peroxidise was blocked by incubation in 3% hydrogen peroxide for 20 minutes. Arrays were then incubated in normal horse serum 1:10 for 30 minutes at 25°C to block non- specific binding. The primary antibody (Monoclonal mouse anti-human, CD34+ Class II, Clone QBEnd 10, Code M7165, DAKO, Glostrup, Denmark) dilutied 1:150 was applied for 30 minutes at 25°C with detection using Envision (DAKO code K5007, Glostrup, Denmark) and DAB (3-3′ diaminobenzidine, Vector code SK 4001, USA) according to manufacturer’s instruction. Arrays were lightly counterstained with haematoxylin, dehydrated and mounted in DPX. Sections of tonsil were used as positive and negative controls for each staining batch as tonsil has many CD34+ cells.

### Slide scanning and scoring

Stained slides were scanned using a Hamamatsu NanoZoomer (Hertfordshire, UK). Visual counting of vessel was performed on a computer monitor.

### Lymphovascular invasion

Lymphovascular invasion was assessed by using a hematoxylin and eosin on full sections. Blood and lymphatic vessel invasion was not distinguished. Lymphovascular invasion was recorded as present or absent.

### Assessment of tumour microvessel density

Microvessel counting was a modification of the method described by Weidner et al. (1992) in which large microvessels as well as any single stained endothelial cells clearly separated from adjacent microvessels, tumour cells, and other connective tissue elements were considered single, countable microvessels. Branching structures were counted as one, unless there was a break in continuity, in which case it was counted as two distinct vessels [37].

The mean vessel count for the three cores of each tumour sample was used for subsequent analysis. One hundred eighty nine cores, counted independently by two observers (C.O. and Z.M.) blinded to patient outcome and the other observer’s score, yielded an interclass correlation coefficient (ICCC) of 0.96, indicating excellent agreement. Z.M. subsequently scored all slides. Accuracy of scoring depends on individual cores containing a satisfactory sample of tumour cells, which was checked by a qualified pathologist (J.J.G.). As there are no universally accepted prognostic thresholds for microvessel counts (CD34+), survival analysis was undertaken using tertiles. In the full-face tissue sections, CD34+ was counted in three x40 fields and the mean for the three fields was calculated. The mean for the three fields in full-face sections was compared with the mean of three cores in TMA for the same patients. The ICCC was 0.87, which indicates excellent agreement.

### Statistical analysis

Inter-relationships between variables were assessed using contingency tables with the chi-squared test for trend as appropriate. Univariate analysis and multivariate survival analysis with calculation of hazard ratios (HR) were performed using Cox’s proportional-hazards model. A stepwise backward procedure was used to derive a final model of the variables that had a significant independent relationship with survival. Deaths up to March 2010 were included in the analysis. Inter-relationships between methods were assessed using contingency table analysis with the chi-squared test for trend as appropriate. Because of the number of statistical comparisons, a *P* value <0.01 was considered to be significant All statistical analysis was performed using SPSS software version 19 (SPSS Inc., Chicago, IL, USA).

## Results

The clinicopathological characteristics of 384 patients with primary operable breast cancer are shown in Table 1.

**Table 1:** The clinico-pathological characteristics of patients with primary operable invasive ductal breast cancer (n = 384)

**Clinico-pathological characteristics**	**Patients (n)**
Age (≤50/>50 years)	107 (28%)/277 (72%)
Size (≤20/21-50/≥50 mm)	218 (57%)/154 (40%)/11 (3%)
Grade (I/II/III)	70 (18%)/157 (41%)/157 (41%)
Involved lymph node (0/1-3/>3)	205 (53%)/108 (28%)/66 (17%)
Oestrogen -receptor status (ER-/ER+)	124 (32%)/237 (62%)
Progesterone -receptor status (PR-/PR+)	193 (50%)/170 (44%)
HER-2 status (HER-2 -/HER-2+)	299 (78%)/65 (17%)
Ki-67 status (Low Ki-67/High Ki-67)	272 (71%)/92 (24%)
LVI (Absent/Present)	234 (61%)/150 (39%)
MVD (CD34+) (tertiles 1, 2, 3)	137 (36%)/126 (33%)/106 (28%)
Loco-regional treatment (Lumpectomy + radiotherapy/mastectomy + radiotherapy)	142 (37%)/242 (63%)
Systemic treatment (ER-based treatment) (hormonal/hormonal + chemotherapy/chemotherapy/none)	192 (50%)/86 (22%)/80 (21%)/22 (6%)

The relationship between ER status and clinico-pathological characteristics is shown in Table 2. Patients with ER negative tumours were younger (*P* < 0.01), had larger carcinomas (*P* < 0.01), of higher tumour grade (*P* < 0.001), more likely to be PR negative tumours (*P* < 0.001), more likely to be HER-2 positive tumours (*P* < 0.001), with more lymphovascular invasion (*P* < 0.001). They were also more likely to receive systemic adjuvant treatment in the form of chemotherapy (*P* < 0.001) and had a shorter cancer- specific survival (*P* < 0.001).

**Table 2:** The relationship between clinico-pathological characteristics and ER status of patients with primary operable invasive ductal breast cancer

	**Oestrogen receptor negative (n = 124)**	**Oestrogen receptor positive (n = 237)**	**( *P *-value)**
Age (≤50/>50 years)	43/81	52/185	0.009
Size (≤20/21-50/>50 mm)	57/62/4	149/83/5	0.004
Grade (I/II/III)	3/22/99	61/125/51	<0.001
Involved lymph node (0/1-3/>3)	65/30/28	130/68/35	0.211
Progesterone -receptor status (PR-/PR+)	117/5	72/161	<0.001
HER-2 status (HER-2 -/HER-2+)	80/41	212/24	<0.001
Ki-67 status (Low Ki-67/High Ki-67)	99/24	160/68	0.036
LVI (Absent/Present)	56/68	161/76	<0.001
MVD (CD34+) (tertiles 1, 2, 3)	38/41/44	83/84/61	0.106
Loco-regional treatment (Lumpectomy + radiotherapy/mastectomy + radiotherapy)	44/80	89/148	0.699
Systemic treatment (ER-based treatment) (hormonal/hormonal + chemotherapy/chemotherapy/none)	26/19/66/11	158/61/8/8	<0.001
Cancer specific survival (months)^*^	130 (118–142)	156 (150–162)	0.001

^*^Mean (95% CI).

The minimum follow-up was 142 months; the median follow-up of the survivors was 165 months. In the patients with ER negative tumours 58 patients developed recurrence, 8 local, 38 distant and 4 both, 79 patients died, 48 of their disease. In the ER positive tumours, 46 patients developed recurrence, 9 local, 31 distant and 1 both, 120 patients died, 47 of their disease.

The relationship between clinico-pathological characteristics of patients with ER negative primary operable invasive ductal breast cancer and recurrence- free survival was examined. On univariate survival analysis tumour size (HR 2.70, 95% CI 1.43-5.09, *P =* 0.002), lymph node involvement (HR 1.71, 95% CI 1.17-2.50, *P =* 0.006), lymphovascular invasion (HR 2.71, 95% CI 1.34-5.50, *P =* 0.006), microvessel density (HR 1.57, 95% CI 1.06-2.34, *P =* 0.026) and local-regional treatment (HR 2.29, 95% CI 1.08-4.87, *P =* 0.032) were significantly associated with recurrence- free survival. On multivariate survival analysis, tumour size (HR 2.27, 95% CI 1.19-4.36, *P =* 0.013) and lymphovascular invasion (HR 2.35, 95% CI 1.10-5.01, *P =* 0.028) were independently associated with recurrence- free survival.

The relationship between clinicopathological characteristics of patients with ER negative primary operable invasive ductal breast cancer and cancer- specific survival is shown in Table 3. On univariate survival analysis tumour size (*P* < 0.05), lymph node involvement (*P* < 0.01), lymphovascular invasion (*P* < 0.05), microvessel density (*P* < 0.05) and loco-regional treatment (*P* < 0.01) were significantly associated with cancer- specific survival. On multivariate survival analysis, lymph node involvement (*P* < 0.01) and loco-regional treatment (*P* < 0.05) were independently associated with cancer- specific survival.

**Table 3:** The relationship between clinico-pathological characteristics of patients with ER negative primary operable invasive ductal breast cancer and cancer- specific survival

	** Univariate survival analysis**		** Multivariate survival analysis**	
	** Cancer-specific survival**		** Cancer-specific survival**	
	** Hazard ratio**	***P*-value**	** Hazard ratio**	***P*-value**
	** (95% CI)**		** (95% CI)**	
Age (<50/>50 years)	0.78 (0.42-1.47	0.448		
Size (≤20/21-50/>50 mm)	2.19 (1.14-4.22)	0.018		0.116
Grade (I/II/III)	1.30 (0.63-2.72)	0.478		
Involved lymph node (0/1-3/>3)	1.85 (1.24-2.66)	0.001	1.71 (1.17-2.50)	0.006
Progesterone -receptor status (PR-/PR+)	0.05 (0.00-22.73)	0.330		
HER-2 status (HER-2 -/HER-2+)	1.26 (0.66-2.41)	0.489		
Ki-67 status (Low Ki-67/High Ki-67)	1.48 (0.70-3.11)	0.305		
LVI (Absent/Present)	2.14 (1.08-4.22)	0.029		0.428
MVD (CD34+) (tertiles 1, 2, 3)	1.62 (1.08-2.44)	0.021		0.130
Loco-regional treatment (Lumpectomy + radiotherapy/mastectomy + radiotherapy)	3.27 (1.44-7.42)	0.005	2.64 (1.14-6.09)	0.023
Systemic treatment (ER-based treatment) (hormonal/hormonal + chemotherapy/chemotherapy/none)	1.00 (0.70-1.42)	1.00		

The relationship between clinicopathological characteristics of patients with ER positive primary operable invasive ductal breast cancer and recurrence- free survival was examined. On univariate survival analysis, tumour grade (HR 1.89, 95% CI 1.12-3.17, *P =* 0.017), lymph node involvement (HR 2.42, 95% CI 1.55-3.76, *P* < 0.001), HER-2 status (HR 2.90, 95% CI 1.25-6.72, *P =* 0.013), Ki-67 proliferative activity (HR 3.01, 95% CI 1.50-6.04, *P =* 0.002), lymphovascular invasion (HR 3.83, 95% CI 1.89-7.77, *P* ≤ 0.001), microvessel density (HR 1.70, 95% CI 1.05-2.73, *P =* 0.030) and systemic treatment (HR 1.59, 95% CI 1.09-2.32, *P =* 0.017) were significantly associated with recurrence- free survival. On multivariate survival analysis, lymph node involvement (HR 2.15, 95% CI 1.35-3.44, *P =* 0.001) and Ki-67 proliferative activity (HR 3.49, 95% CI 1.51-8.07, *P =* 0.003) were independently associated with recurrence- free survival.

The relationship between clinicopathological characteristics of patients with ER positive primary operable invasive ductal breast cancer and cancer- specific survival is shown in Table 4. On univariate survival analysis tumour size (*P* < 0.01), tumour grade (*P* < 0.05), lymph node involvement (*P* < 0.001), Ki-67 proliferative activity (*P* < 0.001), lymphovascular invasion (*P* < 0.001) and systemic treatment (*P* < 0.05) were significantly associated with cancer- specific survival. On multivariate survival analysis, lymph node involvement (*P* < 0.01), Ki-67 proliferative activity (*P* < 0.001) and lymphovascular invasion (*P* < 0.05) were independently associated with cancer- specific survival.

**Table 4:** The relationship between clinico-pathological characteristics of patients with ER positive primary operable invasive ductal breast cancer and cancer- specific survival

	** Univariate survival analysis**		** Multivariate survival analysis**	
	** Cancer-specific survival**		** Cancer-specific survival**	
	** Hazard ratio**	***P*-value**	** Hazard ratio**	***P*-value**
	** (95% CI)**		** (95% CI)**	
Age (<50/>50 years)	1.82 (0.77-4.33)	0.173		
Size (≤20/21-50/>50 mm)	2.04 (1.20-3.49)	0.009		0.116
Grade (I/II/III)	1.71 (1.09-2.68)	0.019		0.342
Involved lymph node (0/1-3/>3)	2.26 (1.54-3.32)	<0.001	2.11 (1.40-3.20)	<0.001
Progesterone -receptor status (PR-/PR+)	0.72 (0.38-1.37)	0.316		
HER-2 status (HER-2 -/HER-2+)	2.17 (0.96-4.88)	0.062		0.109
Ki-67 status (Low Ki-67/High Ki-67)	3.71 (1.98-6.96)	<0.001	3.62 (1.88-7.00)	<0.001
LVI (Absent/Present)	2.98 (1.62-5.47)	<0.001	2.36 (1.22-4.58)	0.011
MVD (CD34+) (tertiles 1, 2, 3)	1.41 (0.95-2.08)	0.087		0.667
Loco-regional treatment (Lumpectomy + radiotherapy/mastectomy + radiotherapy)	1.75 (0.90-3.43)	0.100		
Systemic treatment (ER-based treatment) (hormonal/hormonal + chemotherapy/chemotherapy/none)	1.45 (1.03-2.05)	0.033		0.481

The inter-relationships between clinicopathological characteristics for patients with ER negative primary operable invasive ductal breast cancer are shown in Table 5. Age was negatively associated PR status (*P* < 0.01). Increased tumour size was positively associated with more involved lymph node (*P* < 0.01). Involved lymph node was positively associated with the presence of lymphovascular invasion (*P* < 0.001). HER-2 status was positively associated with the presence of lymphovascular invasion (*P* < 0.01).

**Table 5:** Inter-relationships between the clinicopathological characteristics in patients with ER negative primary operable invasive ductal breast cancer (n = 124)

	** Size**	** Grade**	**Involved lymph node**	**PR status**	**HER-2 status**	**Ki-67 status**	** LVI**	** MVD**	**Loco-regional treatment**	**Systemic treatment**
	**( *P *-value)**	**( *P *-value)**	**( *P *-value)**	**( *P *-value)**	**( *P *-value)**	**( *P *-value)**	**( *P *-value)**	**( *P *-value)**	**( *P *-value)**	**( *P *-value)**
Age (≤50/>50 years)	0.241	0.908	0.242	**0.002**	0.154	0.772	0.826	0.347	0.282	0.059
Size (≤20/21-50/>50 mm)		0.181	**0.004**	0.131	0.343	0.085	0.011	0.106	0.042	0.151
Grade (I/II/III)			0.127	0.049	0.730	0.586	0.042	0.586	0.980	0.164
Involved lymph node (0/1-3/>3)				0.166	0.058	0.392	**<0.001**	0.068	0.013	0.118
Progesterone –receptor status (PR-/PR+)					0.769	0.985	0.111	0.191	0.093	0.272
HER-2 status (HER-2 -/HER-2+)						0.949	**0.001**	0.340	0.068	0.292
Ki-67 proliferative activity (Low Ki-67/High Ki-67)							0.739	0.651	0.256	0.335
LVI (Absent/Present)								0.414	0.054	0.563
MVD (tertiles 1, 2, 3)									0.020	0.944
Loco-regional treatment (Lumpectomy + radiotherapy/mastectomy + radiotherapy)										0.432

Bold data reflects an association between the two variables.

The inter-relationships between clinicopathological characteristics for patients with ER positive primary operable invasive ductal breast cancer are shown in Table 6. Age was negatively associated receiving systemic treatment (*P* < 0.001). Increased tumour size was positively associated with more involved lymph node (*P* < 0.001), the presence of lymphovascular invasion (*P* < 0.001), and loco-regional treatment (*P* < 0.001). Higher tumour grade was positively associated with HER-2 + status (*P* < 0.001), Ki-67 proliferative activity (*P* < 0.001) and the presence of lymphovascular invasion (*P* < 0.001). Involved lymph node was positively associated with the presence of lymphovascular invasion (*P* < 0.001) and loco-regional treatment (*P* < 0.01). The presence of PR was negatively associated with HER-2 + status (*P* < 0.01). Ki-67 proliferative activity was positively associated with microvessel density (*P* < 0.01).

**Table 6:** Inter-relationships between the clinicopathological characteristics in patients with ER positive primary operable invasive ductal breast cancer (n = 237)

	** Size**	** Grade**	**Involved lymph node**	**PR status**	**HER-2 status**	**Ki-67 status**	** LVI**	** MVD**	**Loco-regional treatment**	**Systemic treatment**
	**( *P *-value)**	**( *P *-value)**	**( *P *-value)**	**( *P *-value)**	**( *P *-value)**	**( *P *-value)**	**( *P *-value)**	**( *P *-value)**	**( *P *-value)**	**( *P *-value)**
Age (≤50/>50 years)	0.058	0.236	0.495	0.297	0.054	0.641	0.913	0.238	0.261	**<0.001**
Size (≤20/21-50/>50 mm)		0.033	**<0.001**	0.879	0.826	0.041	**<0.001**	0.568	**<0.001**	0.438
Grade (I/II/III)			0.258	0.753	**<0.001**	**<0.001**	**<0.001**	0.072	0.108	0.627
Involved lymph node (0/1-3/>3)				0.157	0.323	0.035	**<0.001**	0.607	**0.001**	0.013
Progesterone –receptor status (PR-/PR+)					**0.002**	0.340	0.392	0.997	0.256	0.804
HER-2 status (HER-2 -/HER-2+)						0.020	0.119	0.627	0.363	0.603
Ki-67 proliferative activity (Low Ki-67/High Ki-67)							0.049	0.041	0.598	0.289
LVI (Absent/Present)								0.215	0.031	0.255
MVD (tertiles 1, 2, 3)									0.628	0.402
Loco-regional treatment (Lumpectomy + radiotherapy/mastectomy + radiotherapy)										0.057

Bold data reflects an association between the two variables.

## Discussion

The results of the present study showed that lymphovascular invasion but not microvessel density was consistently associated with poorer recurrence- free and cancer-specific survival in both ER negative and ER positive tumours. The results of the present study also confirmed that established tumour characteristics such as tumour size, grade, nodal status, hormone status and Ki-67 proliferative activity provide prognostic value. Therefore, in the context of the present comprehensive examination of the prognostic value of tumour pathological features, it can be concluded that lymphovascular invasion may have an important role in determining outcome in patients with primary operable invasive ductal breast cancer.

The results of the present study are consistent with the previous studies that reported prognostic value of the lymphovascular invasion independent of involvement lymph node as well as other tumour characteristics such as grade, PR and HER-2 status [19-22,25]. However, some studies reported that the presence of LVI was not independently associated with outcome in primary breast cancer [28,48] and others reported no association [49,50].

The results of the present study are also consistent with the previous studies that reported an association between the microvessel density and poorer survival [16,23,32-38]. However, in the present study it was of interest that microvessel density was associated with poorer survival in ER negative but not ER positive tumours whereas lymphovascular invasion was associated with poorer survival in both ER negative and ER positive tumours. Therefore, it would appear that lymphovascular invasion process is more consistently associated with poor outcome in patients with primary breast cancer.

The results of the present study are consistent with the Hayes and co-workers that concluded microvessel density alone could not be recommended as a basis for clinical decision making [51]. However, Kato and co- workers (2003) concluded that microvessel density, by various methods, did offer additional prognostic value to lymphovascular invasion and provided more reliable prognostic information than lymph node status [24].

In this context, it was of interest that in the present study there was an association between increased lymphovascular invasion and lymph node involvement in both ER negative and ER positive tumours. Also, in patients with ER positive tumours there was an association between lymphovascular invasion, tumour size and tumour grade. In contrast, angiogenesis (MVD) was only associated with Ki-67 proliferative activity. Taken together, these results are consistent with the hypothesis a proliferating tumour promotes angiogenesis, lymphovascular invasion, nodal involvement and metastases and then subsequent poor survival. Therefore, it may be that angiogenesis is essential for tumour growth whereas lymphovascular invasion is essential for the tumour to metastasise. Consistent with this hypothesis, in the smallest tumours (<10 mm, n = 66) angiogenesis was observed in 83% of patients and lymphovascular invasion was observed in 29% of patients. In contrast, nodal involvement was only present in 23% of patients. The basis of these associations of lymphovascular invasion, angiogenesis and poorer cancer outcome in ductal breast cancer is not clear but may involve the tumour inflammatory cell infiltrate [52]. Therefore, further work is required to examine these potential relationships. Irrespective, it is apparent that lymphovascular invasion and angiogenesis are not isolated pathological features but are strongly related to other aggressive tumour characteristics. To minimize the risk of measurement bias, two independent observers examined the same cores before assigning an overall score. High levels of independent inter-observer agreement in the assessment of CD34+ suggest that this technique is reliable.

There is limitation of whether a small core sample used in the present TMA was representative of the entire tumour was examined, using a pilot comparison study of at 33 full-faced breast cancer tissue sections.

## Conclusions

In conclusion, lymphovascular invasion and to a lesser extent microvessel density add prognostic value to the established clinical pathological features in patients with primary operable invasive breast cancer.

## Competing interests

The authors declare that they have no competing interests.

## Authors’ contributions

The authors’ responsibilities were as follows- ZMAM did the work and wrote the manuscript: DCMCM and JJG provided significant intellectual input and advice in the statistical analysis: DCMCM, JJG and JE provided significant intellectual input and advice in the writing of manuscript: JD prepared the basic database: EM and CO marked the slides and made TMA. All authors read and approved the final manuscript.

## Pre-publication history

The pre-publication history for this paper can be accessed here:

http://www.biomedcentral.com/1472-6890/13/31/prepub
